# Neuropathy due to lead poisoning

**DOI:** 10.4103/0972-2327.61287

**Published:** 2010

**Authors:** Viroj Wiwanitkit

**Affiliations:** Wiwanitkit House, Bangkhae, 10160, Bangkok, Thailand 10160. E-mail: wviroj@yahoo.com

Sir,

I read the article by Shobha *et al*. with a great interest.[1] I agree that the five indexed cases do indeed have evidence of radial neuropathy. However, I have some concern whether the neuropathy is really the outcome of lead exposure or whether it is due to heavy usage of the wrists during work. Have the authors compared the findings in the patients with that in a matched control group from the same working place? Indeed, in a bigger series of lead poisoning cases from another developing country, the authors have considered this possibility in such kinds of neuropathy.[2]
